# Delayed afterdepolarization‐induced triggered activity in cardiac purkinje cells mediated through cytosolic calcium diffusion waves

**DOI:** 10.14814/phy2.14296

**Published:** 2019-12-23

**Authors:** Chirag Shah, Sohel Jiwani, Bijay Limbu, Seth Weinberg, Makarand Deo

**Affiliations:** ^1^ School of Medicine Eastern Virginia Medical School Norfolk Virginia; ^2^ Department of Engineering Norfolk State University Norfolk Virginia; ^3^ Department of Biomedical Engineering Virginia Commonwealth University Richmond Virginia; ^4^ Department of Biomedical Engineering The Ohio State University Columbus Ohio

**Keywords:** calcium diffusion, cardiac Purkinje cell, delayed afterdepolarizations, triggered activity

## Abstract

Cardiac Purkinje cells (PCs) are more susceptible to action potential abnormalities as compared to ventricular myocytes (VMs), which could be associated with their distinct intracellular calcium handling. We developed a detailed biophysical model of a mouse cardiac PC, which importantly reproduces the experimentally observed biphasic cytosolic calcium waves. The model includes a stochastic gating formulation for the opening and closing of ryanodine receptor (RyR) channels, simulated with a Monte Carlo method, to accurately reproduce cytosolic calcium wave propagation and the effects of spontaneous calcium release events. Simulations predict that during an action potential, smaller cytosolic calcium wavelets propagated from the sarcolemma towards the center of the cell and initiated larger magnitude cell‐wide calcium waves via a calcium‐induced‐calcium release mechanism. In the presence of RyR mutations, frequent spontaneous calcium leaks from sarcoplasmic reticulum (SR) initiated calcium waves, which upon reaching the cell periphery produced delayed afterdepolarizations (DADs) via sodium‐calcium exchanger (NCX) and T‐type calcium (I_CaT_) channel activation. In the presence of isoproterenol‐mediated effects, DADs induced triggered activity by reactivation of fast sodium channels. Based on our model, we found that the activation of either L‐type calcium channels (I_CaL_), I_CaT_, sodium‐potassium exchanger (I_NaK_) or NCX is sufficient for occurrence of triggered activity; however, a partial blockade of I_CaT_ or I_NaK_ is essential for its successful termination. Our modeling study highlights valuable insights into the mechanisms of DAD‐induced triggered activity mediated via cytosolic calcium waves in cardiac PCs and may elucidate the increased arrhythmogeneity in PCs.

## Graphical abstract

Cardiac Purkinje cells (PCs) are more susceptible to action potential abnormalities than ventricular myocytes (VMs), which could be associated with their distinct intracellular calcium handling. We utilized a detailed biophysical model of a murine cardiac PC, capable of producing cytosolic calcium diffusion waves, to study the effects of altered calcium homeostasis in PCs. Our modeling study highlights valuable insights into the mechanisms of triggered activity mediated via cytosolic calcium waves in PCs and may elucidate the increased arrhythmogeneity in PCs.

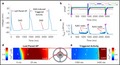

## 1 INTRODUCTION

The cardiac conduction system in ventricles is comprised of a distal network of Purkinje cells (PCs), which are specialized in rapid conduction of electrical impulses (Boyden, 2018). The cardiac PCs are morphologically and electrophysiologically distinct from ventricular myocytes (VMs). Unlike VMs, the T‐tubule structure in PCs is significantly more rudimentary or almost nonexistent, (Di Maio, Ter Keurs, & Franzini‐Armstrong, 2007; Sommer & Johnson, 1968) which results in a distinct calcium (Ca) activation process. Specifically, Ca ions entering the PC via sarcolemmal channels (L‐ and T‐ type Ca channels) diffuse through the cytoplasm to reach the sarcoplasmic reticulum (SR) to activate the Ca‐induced‐Ca release process. Indeed, biphasic cytosolic Ca waves have been recorded in isolated PCs as well as Purkinje strands upon electrical stimulation, which consists of low amplitude radial wavelets and larger cell‐wide longitudinal Ca waves (Boyden, Pu, Pinto, & Keurs, 2000; Stuyvers et al., 2005).

It has been well‐established that PCs are more prone to action potential abnormalities such as early‐ and delayed‐ afterdepolarizations (EADs and DADs, respectively) compared to VMs (Herron, Milstein, Anumonwo, Priori, & Jalife, 2010; Szabo, Kovacs, & Lazzara, 1995). DADs may result from Ca‐activated transient inward currents caused by spontaneous Ca release (SCR) from the SR. The increased vulnerability of PCs to DADs has been attributed to higher propensity of SR Ca release and higher diastolic intracellular Ca‐membrane voltage coupling gain (Maruyama et al., 2010). The heterogeneous electrotonic transmission properties at Purkinje‐myocardium junctions may readily facilitate propagation of triggered action potentials in PCs due to stochastic SCR events (Ben Caref, Boutjdir, Himel, & El‐Sherif, 2008; Deo, Boyle, Kim, & Vigmond, 2010; Schafferhofer‐Steltzer, Hofer, Huelsing, Bishop, & Pollard, 2005). Recent experimental studies in transgenic mice carrying ryanodine receptor (RyR2) mutations, typically found in catecholaminergic polymorphic ventricular tachycardia (CPVT) patients, have demonstrated that the Ca‐dependent triggers of arrhythmia may originate in the Purkinje system (Cerrone et al., 2007; Kang et al., 2010; Willis et al., 2016).

Altered Ca dynamics has been shown to be arrhythmogenic, (Santulli & Marks, 2015) but the mechanisms by which cytosolic Ca transients play a role in promoting afterdepolarizations are not clearly understood. The Ca‐mediated triggered activity has been studied before in ventricular myocytes. However, there is a dearth of similar investigations in cardiac PCs. Specifically, a mechanistic link between the distinct calcium homeostasis in PCs to their increased arrhythmogeneity, compared to that in VMs, has not been studied systematically. It has been established that PCs with RyR2 mutations have a lower threshold to develop SCRs and greater propensity for triggered activity. However, the mechanism of PC vulnerability to arrhythmogenic triggers is poorly understood. In this study, we utilize a multiscale modeling approach to implement a spatiotemporal model of cytosolic Ca diffusion waves in mouse PCs produced by stochastic RyR gating formulation. The model was used to study the mechanisms of DAD‐induced triggered activity mediated through cytosolic Ca diffusion waves in the presence of RyR2 mutations.

## 2 METHODS

### 2.1 Biophysical model of mouse PC

**Figure 1:**
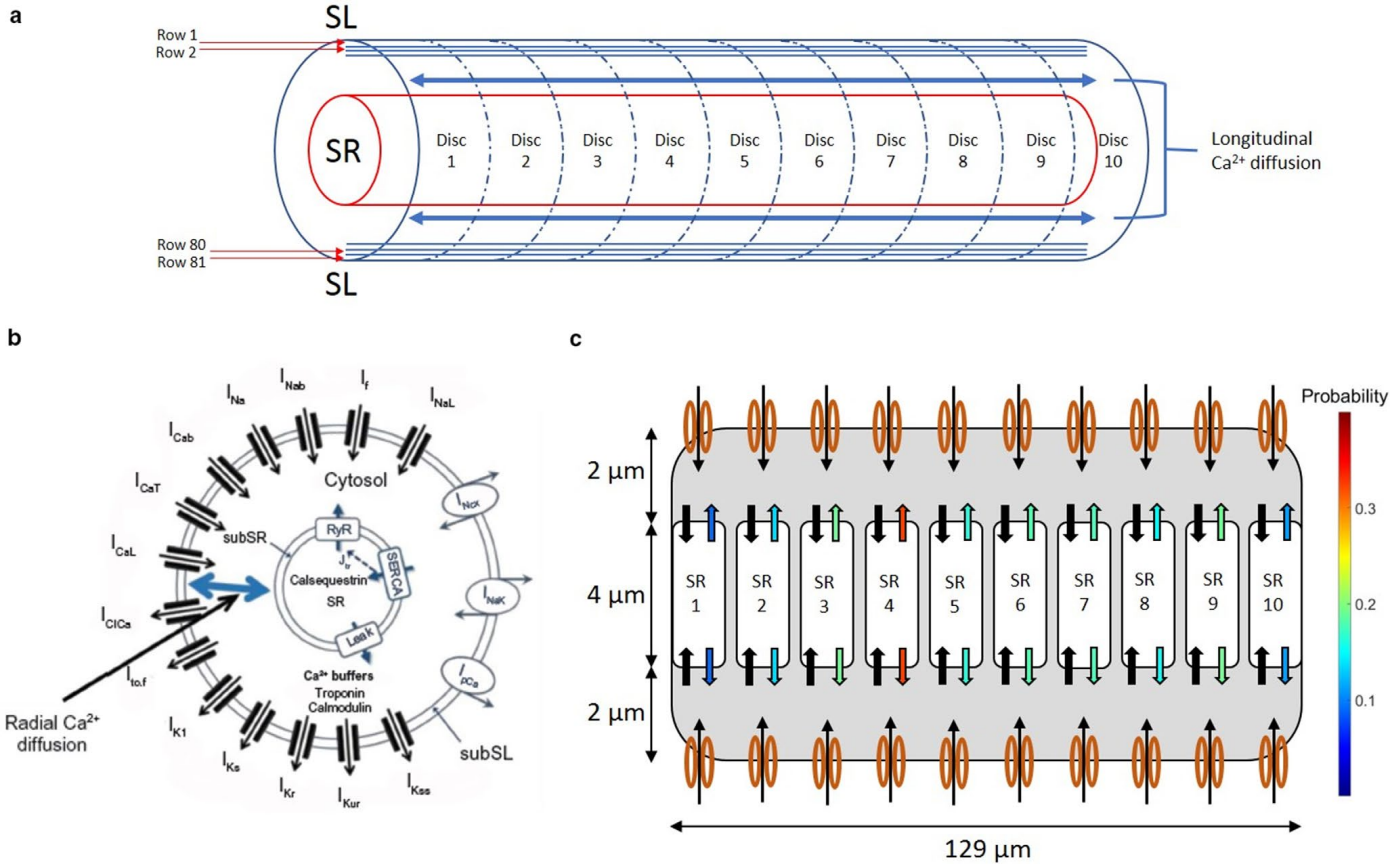
(a) Schematic representation of the PC biophysical model divided into 10 discs and 81 concentric layers showing longitudinal Ca^2+^ diffusion. (b) Cross sectional schematic of the model illustrating ionic currents and illustrating radial Ca^2+^ diffusion. (c) Representation of the SR split into 10 discs, with black arrows representing inward Ca^2+^ movement (Network SR) and colored arrows representing Ca^2+^ efflux from SR (Junctional SR). Arrow colors correspond to RyR open probabilities at a specific time point during stimulus‐induced action potential

Our biophysical model of the mouse PC with simulation of cytosolic Ca diffusion is described in detail elsewhere (Limbu, Shah, Weinberg, & Deo, 2016). Briefly, the model incorporated the late Na^+^ current (I_NaL_) from the study by Li and Rudy (2011) in addition to geometrical modifications to the mouse PC model described by Vaidyanathan et al., (2013) (Figure 1a and b). The model assumes a cylindrical cell with 129 µm length and 8 µm width in accordance with experimental measurements. The length of the cell was discretized into 10 discs and the width into 81 concentric layers. We assume the SR to be distributed along the core of the cell with two separate compartments: (a) the release compartment, called the Junctional SR (JSR), releasing Ca into cytosol, and (b) the uptake compartment, called Network SR (NSR), bringing cytosolic Ca back into the SR. RyR channels involved in the calcium induced calcium release (CICR) process are located within the JSR membrane. The sarcolemmal Ca flux and the RyR channels are linked through radial and longitudinal cytosolic Ca diffusion model (described in Ref. Limbu et al., 2016).

Two significant modifications were made to our previous model (Limbu et al., 2016). Firstly, the radial and longitudinal diffusion of Ca were no longer assumed to be equivalent. Based on analysis of Ca transients by Stuyvers et al. (2005) the longitudinal diffusion coefficient was reduced by 30% to reflect the decreased velocity of cell‐wide Ca waves (CWW) particularly from the SR. Secondly, a stochastic gating model was implemented for the RyR channel states, as described below (Figure 1c).

### 2.2 Stochastic gating of RyR channel – Monte Carlo method

In the absence of spatial heterogeneity information in the Ca release sites, stochastic Ca release is a necessary requirement to simulate propagating longitudinal waves. Otherwise, every release site would be activated at the same time. The stochastic nature of Ca channels and its physiological importance, especially in small subspace volumes has been well‐documented (Rüdiger et al., 2007; Weinberg & Smith, 2012). To account for this stochastic behavior, we employed a stochastic gating model for the opening and closing of RyR channels, simulated using a Monte Carlo method (Smith, 2002).

For a two‐state Markov model with opening and closing rates of *k_open_* and *k_close_*, respectively, the ordinary differential equation for RyR open probability was adopted from Korhonen et al., (2009) as described in Equation (1),

(1) $$\frac{dP_{\text{open}}}{dt}=P_{\text{close}}k_{\text{open}}{\left[1+{\left(K_{\text{m,RyR}}/{\left[{\text{Ca}}^{2+}\right]}_{\text{subSR}}\right)}^{4}\right]}^{-1}-k_{\text{close}}P_{\text{open}}$$

where *K_m,RyR_* is the half‐saturation concentration of the RyR channel (Boyden, 2018) and is given by Equation (2),

(2) $$K_{\text{m,RyR}}=3.51*{\left[1+\exp\left(\left({\left[{\text{Ca}}^{2+}\right]}_{\text{JSR}}-530\right)/200\right)\right]}^{-1}+0.25$$

For *N* total number of RyR channels per SR disc (total of 10 SR discs), the number of open and closed RyR channels is given by Equations (3) and (4):

(3) $$N_{\text{open}}=N*P_{\text{open}}$$

(4) $$N_{\text{close}}=N*P_{\text{close}}=N-N_{\text{open}}$$

It thus follows that the probability of transitions occurring from closed to open states for the RyR channels during a time interval of duration *dt* is described as:

(5) $$\begin{array}{c} P\left(s=O,t+dt|s=C,t\right\}=P_{\text{T,open}}\\ \;\;\;\;\;\;\;=k_{\text{open}}{\left[1+{\left(K_{\text{m,RyR}}/{\left[{\text{Ca}}^{2+}\right]}_{\text{subSR}}\right)}^{4}\right]}^{-1}*N_{\text{close}}*dt \end{array}$$

Conversely, the probability of transitions occurring from open to closed states is described as:

(6) $$P\left(s=C,t+dt|s=O,t\right\}=P_{\text{T,close}}=k_{\text{close}}*N_{\text{open}}*dt$$

To stochastically update *P_open_* and *P_close_*, a random number *U* uniformly distributed on the interval [0,1] is chosen. *P_open_* and *P_close_* are then updated based on comparisons to *U* that are defined in Equations (7), (8) and (9).

(7) $$\text{If}\;U<PT,\;\text{open}\;\left(\text{channel opening}\right):\;P_{\text{open}}=\frac{N_{\text{open}}+1}{N}$$

(8) $$\text{If}\;P_{\text{T,open}}<U<\left(P_{\text{T,open}}+P_{\text{T,close}}\right)\;\left(\text{channel}\;\text{closing}\right):\;\;P_{\text{open}}=\frac{N_{\text{open}}-1}{N}$$

(9) $$\;\text{If}\;U>\left(P_{T,\text{open}}+P_{T,\text{close}}\right)\left(\text{no change}\right):\;\;P_{\text{open}}=\frac{N_{\text{open}}}{N}$$

This methodology was applied to each of the 10 SR discs for each time step to stochastically update the open probabilities of the RyR channels.

### 2.3 Modeling RyR2 mutation

Aberrant increased release of Ca from RyR channels is a potential mechanism by which RyR2 mutations can result in an arrhythmogenic trigger (Cerrone et al., 2007; Herron et al., 2010). To model this abnormal behavior, a stochastic approach was implemented in which a random number *Y_leak_* uniformly distributed on the interval [0,1] was chosen for each disc and multiplied by SR [Ca] for the respective disc, as described in Equation (10), where *i* represents an individual disc.

(10) $$Y=Y_{\left(\text{leak},i\right)}*{\left[{\text{Ca}}^{\left(2+\right)}\right]}_{\left(\text{JSR},i\right)}$$

Scaling by the SR [Ca] was included to model the process of spontaneous Ca release from RyR channels when the SR is overloaded with Ca, a phenomenon known as store overload‐induced Ca release (SOICR) (Jiang et al., 2005). The threshold value of *Y* was set such that the probability of an RyR channel leak was no greater than one percent when the SR [Ca] was greater than 600 µM, which is an approximate upper limit for SR [Ca] during burst pacing.

If the value of *Y* was sufficient to induce a leak from the RyR channels of a specific disc, the open probability of the RyR channels for that disc was assigned in the following manner: A random number *Z* that followed a log normal distribution with µ = 1‐ln(0.1) ≈−1.3 and σ = .5, chosen to replicate probabilities that were similar to open probabilities during a stimulus‐induced action potential. Any value of *Z* greater than 1 was assigned a probability of 1. For simplicity and to more concisely study the effects of inappropriate Ca release via RyR channels, only one disc had a Ca leak event at a particular time. Furthermore, experimental studies have reported that spontaneous Ca releases are sustained events, leading to significant releases of Ca from the SR (Faber & Rudy, 2007). To model sustained events, a time constant τ, similar to that used in the study by Faber and Rudy (2007) was incorporated into the stochastic Ca release model, with a value of τ = 125 ms.

### 2.4 Isoproterenol stimulation and quantification of Ca^+^ sparks

To simulate the effects of isoproterenol, the maximum conductance of six channels was altered (Faber & Rudy, 2007; Gaur, Rudy, & Hool, 2009; Zhang, Wang, & Nattel, 2002). Conductance through the T‐type Ca channel (I_CaT_) was increased conservatively by 115% since prior studies have reported the effects of isoproterenol to increase its maximum conductance by approximately 100%–130% (Willis et al., 2016; Zhang et al., 2002). Similarly, conductance through the L‐type Ca channel (I_CaL_) was conservatively increased by 100% based on the reported range of 40%–180% (Faber & Rudy, 2007; Gaur et al., 2009; Willis et al., 2016). Maximum conductance in the Na^+^/K^+^ pump (I_NaK_), slow delayed rectifier K^+^ current (I_Ks_), and SR Ca ATPase (SERCA) were upregulated by 35%, 80% and 20%, respectively (Faber & Rudy, 2007). Maximum conductance of the inward rectifier K^+^ channel (I_K1_) was downregulated by 20% (Gaur et al., 2009; Willis et al., 2016).

Analysis of Ca sparks was performed in simulations that allowed for stochastic Ca release from SR, in the presence and absence of isoproterenol. The cell model was paced for 5 s to achieve a steady state, and then the number of sparks occurring during a 15‐s interval post pacing was measured. Quantification of Ca sparks after a release event was done by analyzing the flux through RyR channels for each disc during and after the release event. All Ca sparks that were sustained for at least 1 ms were considered. In the absence of isoproterenol, distinct changes in the flux through RyR channels for each disc were readily observed, and all sparks were counted if the 1 ms duration condition was satisfied. In the presence of isoproterenol, distinct changes in RyR flux were not readily observed, as there was significant variability in the RyR flux. To quantify these sparks, a methodology adapted from Cheng et al. (Smith, 2002) was employed. Briefly, the flux through RyR channels during release events was analyzed for each disc, and, as mentioned previously, all sustained releases of 1 ms were considered. Noise was eliminated by choosing the amplitude of changes in flux that were two standard deviations above the mean during the 15 s postpacing. These points were then removed from the data and the mean and standard deviation of the amplitude of changes in flux were recalculated, with values exceeding two standard deviations above the new mean being chosen from this new set of points. This approach allowed for removal of any potential outliers that may skew the overall mean and standard deviation. Data from these two sets of points were combined to provide the total number of Ca sparks.

Simulations were performed with stimulation frequencies of 1–20 Hz, with a stimulus current of amplitude −80 µA/µF and duration of 0.5 ms. Prior to analysis, the model was paced at each frequency for 5 min to obtain steady state conditions. Following the experimentally established protocol to overload the SR with Ca, (Cerrone, Napolitano, & Priori, 2009; Herron et al., 2010; Kang et al., 2010; Liu et al., 2006; Sedej et al., 2010; Willis et al., 2016) the RyR2‐mutated models were burst paced at 5, 15 and 20 Hz. Membrane voltage, ion channel currents, and RyR channel gating and flux were measured during the following 15 s postpacing.

## 3 RESULTS

### 3.1 Action potential morphology and stochastic RyR gating

**Figure 2:**
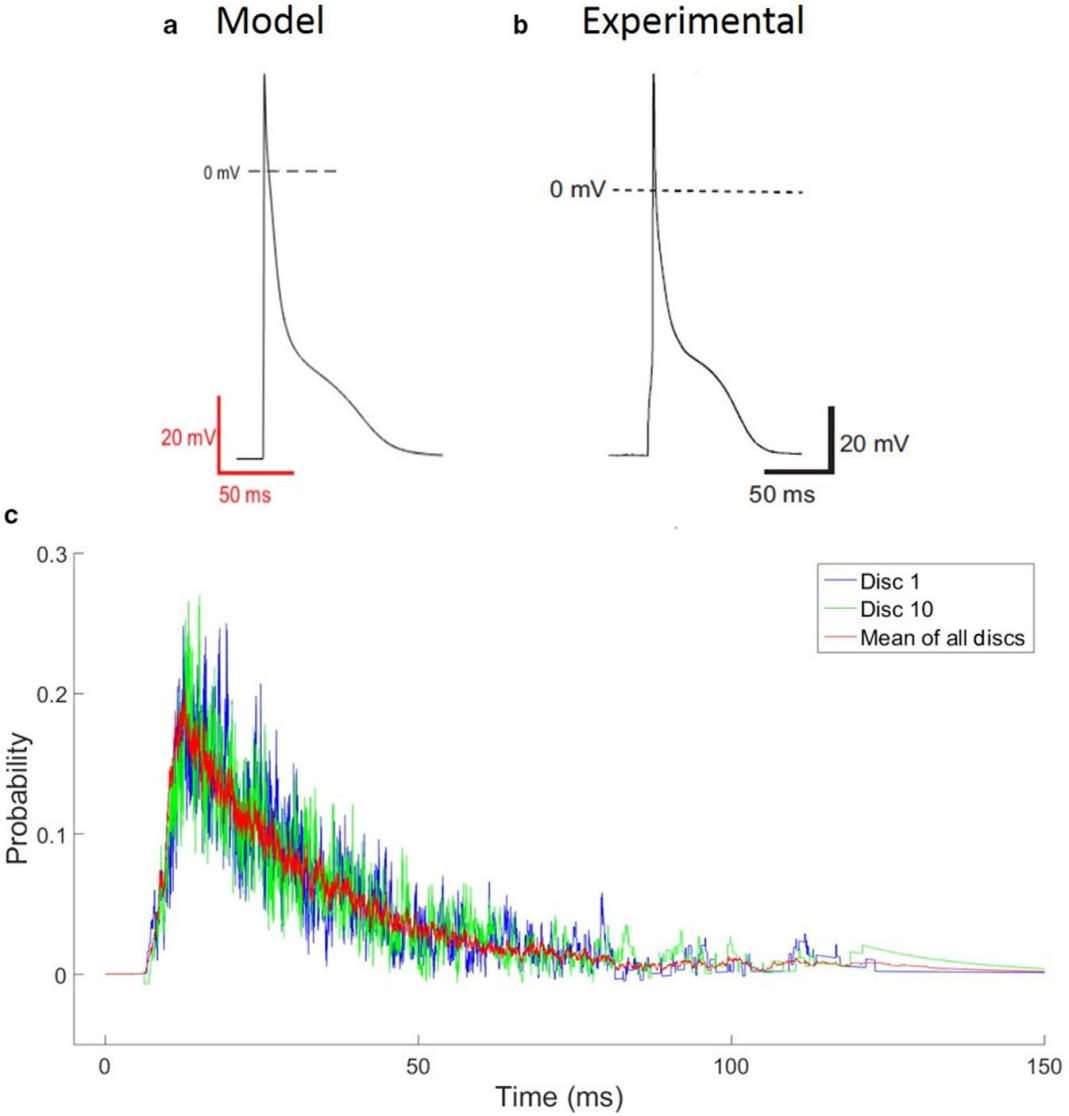
Typical action potential (AP) morphology during stimulation at 1 Hz (a) in our control PC model and (b) experimental recordings (Vaidyanathan et al., 2013). (c) RyR channel open probabilities for typical AP during 1 Hz stimulation in the control PC model, showing tracings from two of the 10 discs and the disc mean

Figure 2 illustrates the AP morphology during 1 Hz stimulation obtained in our model (Figure 2a), in comparison to experimental recordings (Figure 2b) from mouse PCs (Vaidyanathan et al., 2013). We quantified the maximum d*V/*d*t* and AP durations at 70% (APD_70_) and 90% (APD_90_) for the model during 1 Hz stimulation. The stochastic model replicated both APD_90_ and maximum d*V/*d*t* values: Mean APD_90_ in the model was 67.19 ± 1.32 ms, compared to mean APD_90_ of 68.6 ± 5 ms in experiments (Vaidyanathan et al., 2013). Mean d*V/*d*t*_max_ in our model was 223.12 ± 0.03 mV/ms, which was within the range of experimental values (Vaidyanathan et al., 2013). The average Ca transients in our model agree with the experimentally recorded transients in Vaidyanathan et al. (2013) in terms of rise time, decay time and time constant. Table 1 provides a detailed comparison between the model outcome and previously published experimental measurements of AP morphology and average Ca transients. We have previously shown that the distinctive low‐voltage plateau in mouse PCs, ranging between −40 and −60 mV, is due to the effects of T‐type Ca channels (Limbu et al., 2016).

**Table 1:** AP and average Ca transient parameters in experiments (Vaidyanathan et al., 2013) and control model paced at 1 Hz

	Parameter	Experiments (Vaidyanathan et al., 2013)	Model
AP parameters	dV/dt_max_ (mV/ms)	212 ± 15	223.12 ± 0.03
	APD50 (ms)	4.7 ± 0.3	9.3 ± 0.00
	APD70 (ms)	14.4 ± 1.6	19.42 ± 0.07
	APD90 (ms)	68.6 ± 5	67.19 ± 1.32
Ca^2+^ parameters	Peak amplitude	31.193 ± 0.91 AU	0.92 ± 0.01 µM
	Time to peak (ms)	18.8 ± 13.2	23.93 ± 0.67
	Decay time (ms)	261 ± 69.18	208.00 ± 1.91

Note

Ca^2+^ parameters based on average total intracellular Ca^2+^

RyR channel open probabilities are shown during a single stimulus‐induced AP (Figure 2c). For simplicity, only two discs are shown along with the average of all discs. As Ca diffuses from the sarcolemma (SL) to the SR, the RyR channel open probability increases. The volatility of open probabilities over time is indicative of numerous channel gating state transitions. Elevated Ca levels at the SR induce CICR and initiate cell‐wide Ca waves (CWWs). Due to the stochastic nature of the RyR channels, Ca transients from the SR during CICR adopt a similar morphology, as described below.

### 3.2 Ca^2+^ transients and cell‐wide Ca^2+^ waves

**Figure 3:**
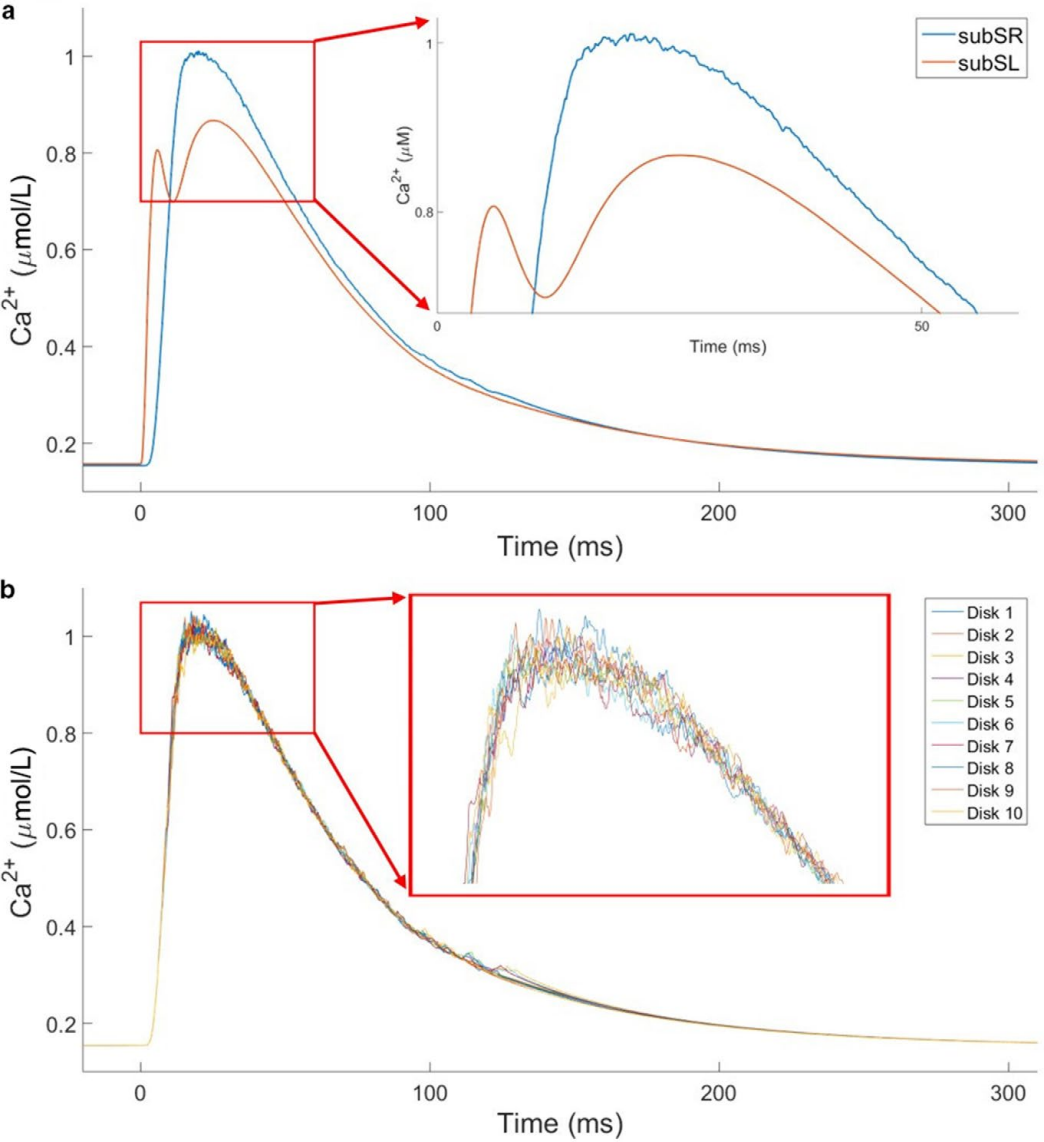
(a) Average Ca^2+^ transients at sub‐SL and sub‐SR regions for a stimulus‐induced AP during 1 Hz stimulation in our control PC model. (b) Sub‐SR Ca^2+^ transients for individual discs for a stimulus‐induced AP during 1 Hz stimulation in our control PC model. Inset images in both (a) and (b) show peak Ca^2+^ transients to highlight stochastic gating of RyR channels

The time course of the average [Ca]_i_ at the SL demonstrates the biphasic nature of Ca transients in the subsarcolemmal (sub‐SL) region, with the sub‐SR region shown for comparison (Figure 3a). The first peak in the sub‐SL Ca time course occurs due to extracellular Ca entering the cell through sarcolemmal Ca channels triggered during an AP. Ca diffuses radially inward and triggers SR Ca release via RyR channels during CICR, giving rise to the sub‐SR Ca peak. Ca diffuses then radially from the core of the PC and reaches the sub‐SL region, giving rise to the delayed second sub‐SL peak. Importantly, we can observe that sub‐SR Ca is clearly elevated prior to the second sub‐SL peak. In order to further study this biphasic nature of Ca transients in the sub‐SL region, we replicated the study with increased pacing rates of 2–5 Hz and 10 Hz. We found that the biphasic nature was consistent event at higher pacing frequencies, though the magnitude of the delayed second sub‐SL peak decreased with increasing pacing frequency. Figure S1 shows action potentials and Ca transients elicited in sub‐SL and subSR regions at 5 Hz, and 10 Hz pacing clearly showing the biphasic nature of cytosolic Ca.

The inset in Figure 3a, specifically the sub‐SR Ca trace, highlights the stochastic release of Ca through RyR channels. A fluctuating sub‐SR [Ca] is evident at the peak, which corresponds to fluctuating Ca releases from the 10 discs (Figure 3b). Since our model integrates stochasticity into each of the 10 discs, the sub‐SR [Ca] at each disc varies over time, with individual discs having a distinct maximum sub‐SR [Ca] (Figure 3b inset).

**Figure 4:**
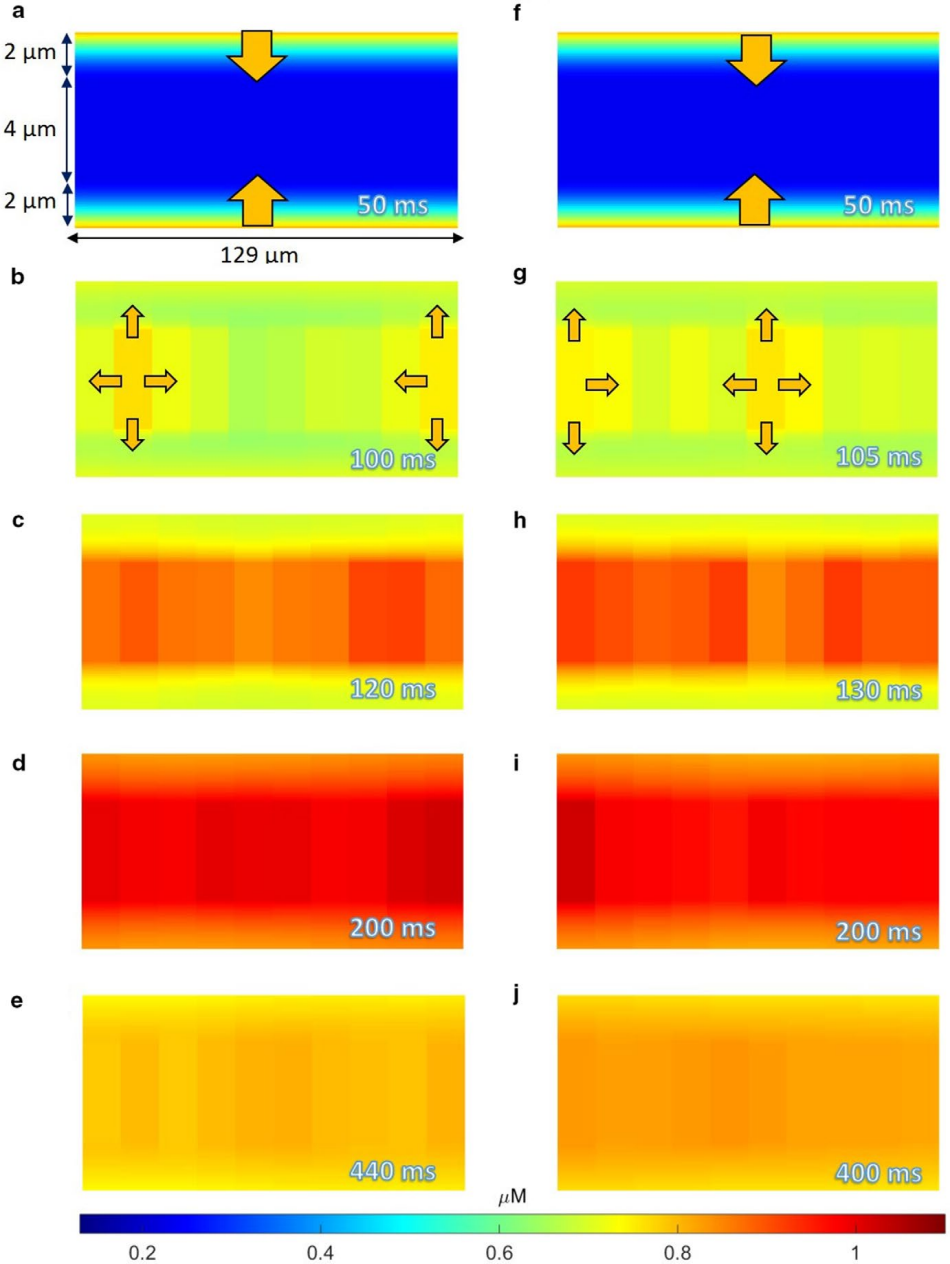
(a–e) Ca^2+^ spatial plots at various times during an AP in the control PC model, with discs 2 and 10 inducing CICR. (f–j) Ca^2+^ spatial plots at various times during a different AP in the control PC model, with discs 1 and 6 inducing CICR. Arrows in (a) and (f) represent movement of extracellular Ca^2+^ into PC, while arrows in (b) and (g) represent movement of SR Ca^2+^ into cytosol

As we have noted previously, Ca wavelets occur due to small increases in sub‐SL [Ca] (Ca influx from sarcolemmal channels), whereas CWWs occur with larger increases in [Ca] from the SR (Boyden et al., 2000). Figure 4 shows two examples of wavelets and CWWs (Panels A–E, and F–J). Wavelets are initially generated in the sub‐SL region when a stimulus‐induced AP causes Ca to enter the PC via SL Ca channels during phase 2, represented by the arrows in Figure 4a and f. As these wavelets continue to develop, cytosolic Ca radially diffuses towards the core, elevating the sub‐SR [Ca] levels. Due to the stochastic nature of RyR channel gating, the location within the cell for CWW initiation is random. This is shown in Figure 4b and g, in which discs 2 and 10 initiate Ca release in the first example (Figure 4b) and discs 1 and 6 initiate Ca release in the second example (Figure 4g). Once CICR is triggered in the initial disc, a large Ca release from the SR of the respective discs initiates the longitudinal diffusion to adjacent discs, in addition to radial diffuse toward the SL. Thus, Ca levels are elevated in adjacent discs from both radial and longitudinal directions, which is sufficient to trigger CICR processes in these discs. This process continues, triggering successive SR Ca releases and driving the CWW to propagate longitudinally, as shown in Figure 4c and h. After Ca release occurs in all discs (Figure 4d and i), SR Ca is depleted and RyR channels close. This leads to termination of the CWW, as shown in Figure 4d–e and i–J. Importantly, Ca released from each disc also propagates radially from the cell core region to the periphery, thus elevating the [Ca] in sub‐SL region as well.

### 3.3 Delayed afterdepolarizations and triggered activity in CPVT

**Figure 5:**
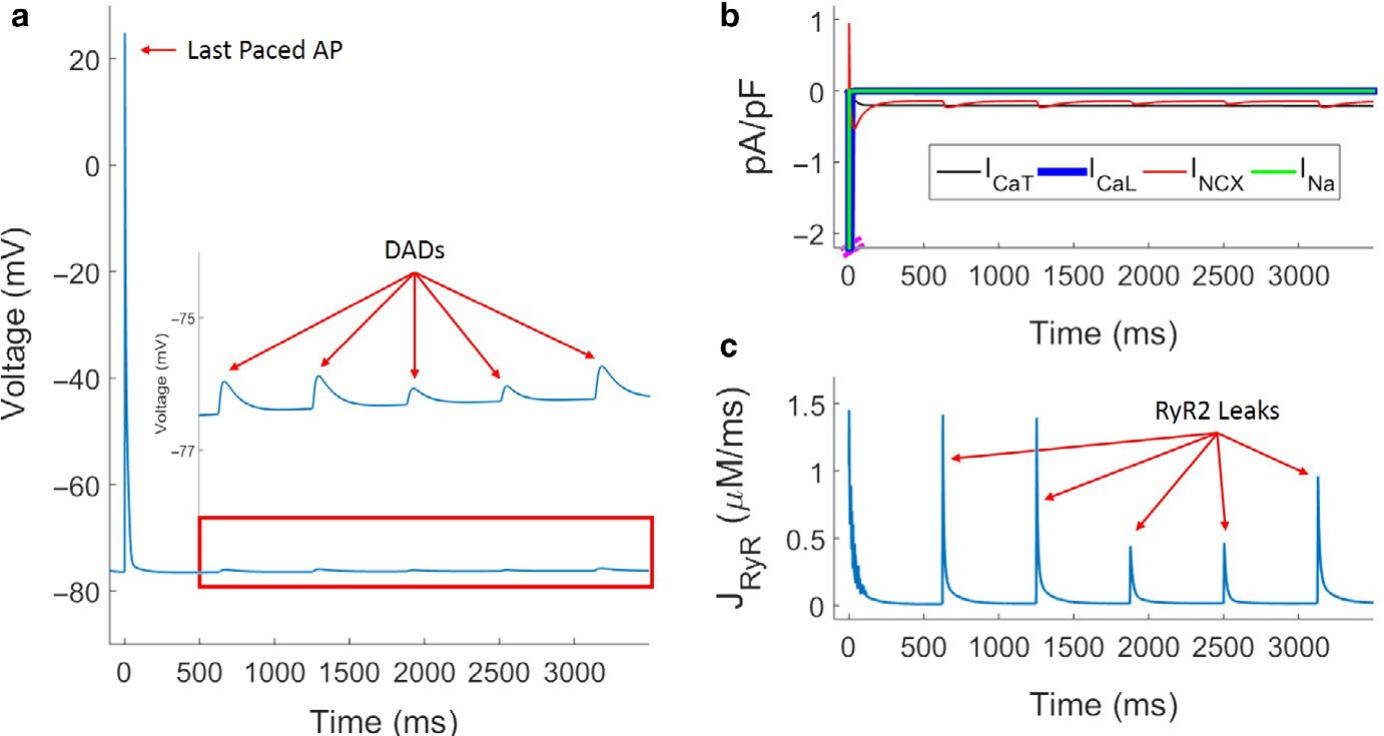
DAD formation in CPVT‐phenotype PCs following burst pacing for 5 s, in the absence of isoproterenol. (a) DADs observed in the membrane voltage postpacing. (b) Membrane currents postpacing driving DAD formation. (c) RyR leak events trigger DAD formation. Time 0 corresponds to the timing of the last paced AP

**Figure 6:**
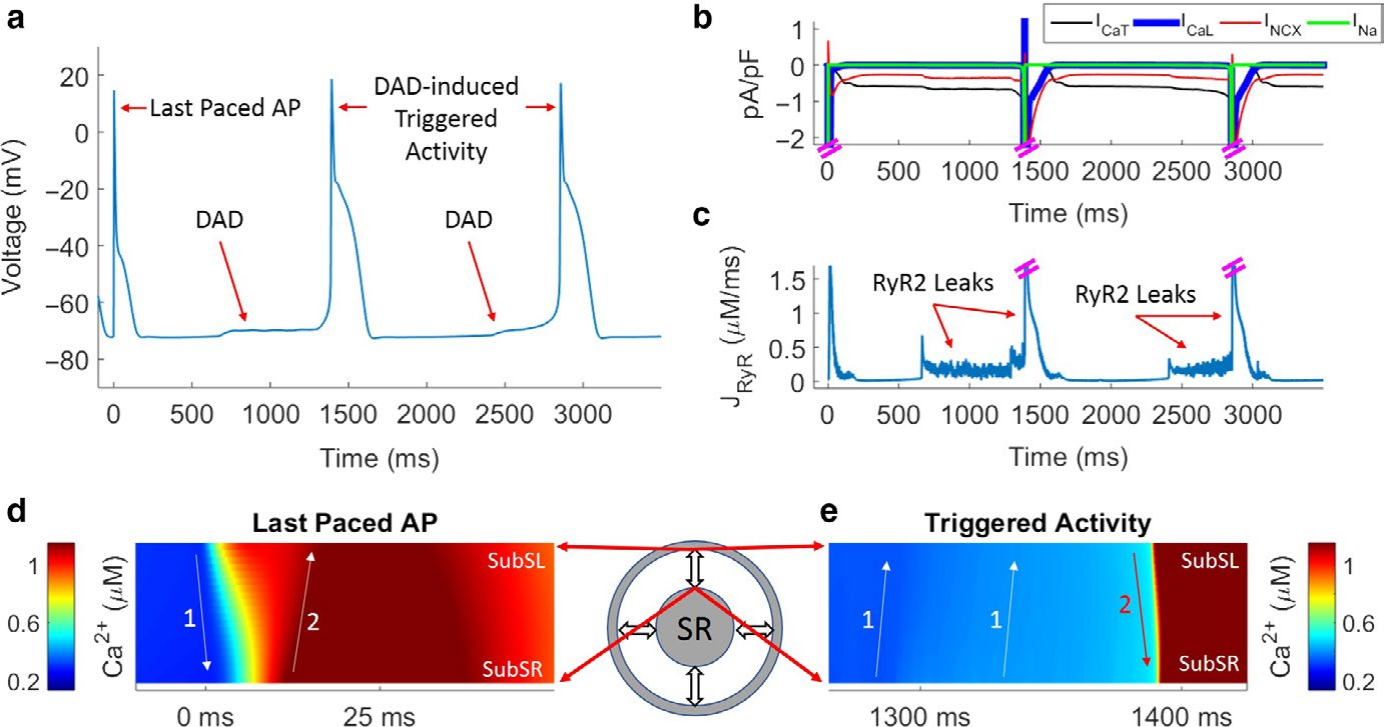
DAD formation in CPVT‐phenotype PCs following burst pacing for 5 s, in the presence of isoproterenol. (a) Membrane voltage postpacing illustrates DADs and triggered activity. (b) Membrane currents postpacing during DADs and triggered activity. (c) RyR leak events drive DADs and triggered activity. (d) [Ca^2+^] from sub‐SL to sub‐SR during the last paced AP. (e) [Ca^2+^] from sub‐SL to sub‐SR during triggered activity. Time 0 corresponds to the timing of the last paced AP

To study the CPVT phenotype and the role of the RyR2 mutation in PCs, we applied burst pacing at 1, 5, 15 and 20 Hz stimulation for 5 s and observed the activity in the following 15 s postpacing. Figures 5 and 6 demonstrate responses following burst pacing at 5 Hz in the absence and presence of isoproterenol‐mediated effects, respectively. In the absence of isoproterenol, several subthreshold DADs occurred in the three seconds postpacing (Figure 5a). Key sarcolemmal ion channel currents and RyR flux are shown in Figure 5b and c, respectively. Figure S2 shows spontaneous triggered activity followed by 20 Hz burst pacing in presence of isoproterenol. We find that SCR events drive the RyR leak fluxes and drive an inward current through the sodium‐calcium exchanger (NCX), which in turn triggers the DADs. The amplitude of the RyR flux and corresponding DAD amplitudes vary due to stochasticity. While DADs were frequently observed in simulations, triggered activity was not observed in the absence of isoproterenol in any simulations (1–20 Hz burst pacing). To further compare the control model and the isoproterenol model, we studied the differences in cytosolic Ca^2+^ and JSR Ca^2+^ between the two models. Average Ca^2+^ values over the 5 s of burst pacing and 15 s postpacing were calculated separately for the two cellular regions. During the 5 s of burst pacing, the average cytosolic Ca^2+^ was 61.55 µM and 106.59 µM in the control and isoproterenol models, respectively. JSR Ca^2+^ during burst pacing was 490.20 µM and 392.39 µM in the control and isoproterenol models, respectively. In the 15 s postpacing, average cytosolic Ca^2+^ was 37.19 µM and 97.05 µM in the control model and isoproterenol models, respectively, while JSR Ca^2+^ was 467.16 µM and 401.88 µM in the control and isoproterenol models, respectively.

Figure 6a–e demonstrate the effects of isoproterenol in our RyR‐mutated model after burst pacing at 5Hz for 5 s, with time 0 indicating the last paced AP. Two DADs are seen in the three seconds postpacing, with both DADs converting into triggered activity (Figure 6a). In the presence of isoproterenol, both I_caT_ and NCX are persistent inward currents following pacing (Figure 6b). Membrane depolarization due to the combined effects of I_caT_ and NCX reached the fast sodium channel activation (I_Na_) threshold, which in turn led to the triggered AP. During triggered activity, ionic currents replicated the changes seen during a stimulus‐induced AP (Figure 6b). RyR channel flux was prolonged during each leak event (SCR), compared to the leak events without isoproterenol (Figure 6c). Additionally, flux through RyR channels during triggered activity was larger than RyR channel flux during a stimulus‐induced AP.

Ca linescan plots demonstrate the differences between the Ca transients during stimulus‐induced APs and triggered activity under the effects of isoproterenol (Figure 6d and e, respectively). Figure 6d shows the expected CICR with biphasic Ca transients. At the start of the AP, Ca moves from the sub‐SL to the sub‐SR (Figure 6d, arrow 1). As these small wavelets diffuse towards the subSR, larger waves are triggered with significant Ca release from the SR (Figure 6d, arrow 2), consistent with the biphasic nature of Ca transients described earlier. However, during triggered activity (Figure 6e), the increase in cytosolic Ca is initiated at the subSR due to an SCR event (Figure 6e, arrow 1), which diffuses radially to the subSL region. As cytosolic [Ca] in subSL region increases, Ca‐driven inward SL currents drive the DAD formation that leads to a triggered AP. A large influx of Ca into the cytosol occurs during the triggered AP and then diffuses towards subSR (Figure 6e, arrow 2). Moreover, cytosolic Ca concentrations reach much higher values during triggered activity than during stimulus‐induced APs. This is due to the increased magnitude and duration of inward currents of both I_CaL_ and NCX during triggered activity (Figure 6b). During triggered activity, inward I_CaL_ reaches a magnitude of 17.8 pA/pF as opposed to 13.8 pA/pF during a stimulus‐induced AP. Similarly, an inward NCX current reaches a magnitude of 2.5 pA/pF during triggered activity, compared to 0.7 pA/pF during a stimulus‐induced AP.

### 3.4 Ca^2+^ sparks in CPVT

We next analyzed Ca sparks, which represent stochastic releases of Ca from RyR channels, in the presence and absence of isoproterenol in the CPVT model. Figure S3 shows Ca plots that demonstrate Ca sparks during DAD‐induced triggered activity in the presence of isoproterenol. A random leak event initiates in Disc 5 (Figure S1a). The arrows highlight discs where Ca sparks are occurring at the specific times noted (with respect to the time of initial leak in Disc 5). Following the initial Ca spark event, subsequent Ca sparks tended to be induced in discs adjacent to the leaking discs; however, this longitudinal spark pattern became less apparent as leaks continued, and a more random spark pattern among the discs was observed (Figure S1b–d). During initiation of DAD‐induced triggered activity, Ca diffusion from the sub‐SL to the sub‐SR, triggered a propagating increase in SR release of Ca along the length of the cell (Figure S1e–f), as described previously in Figure 6.

**Figure 7:**
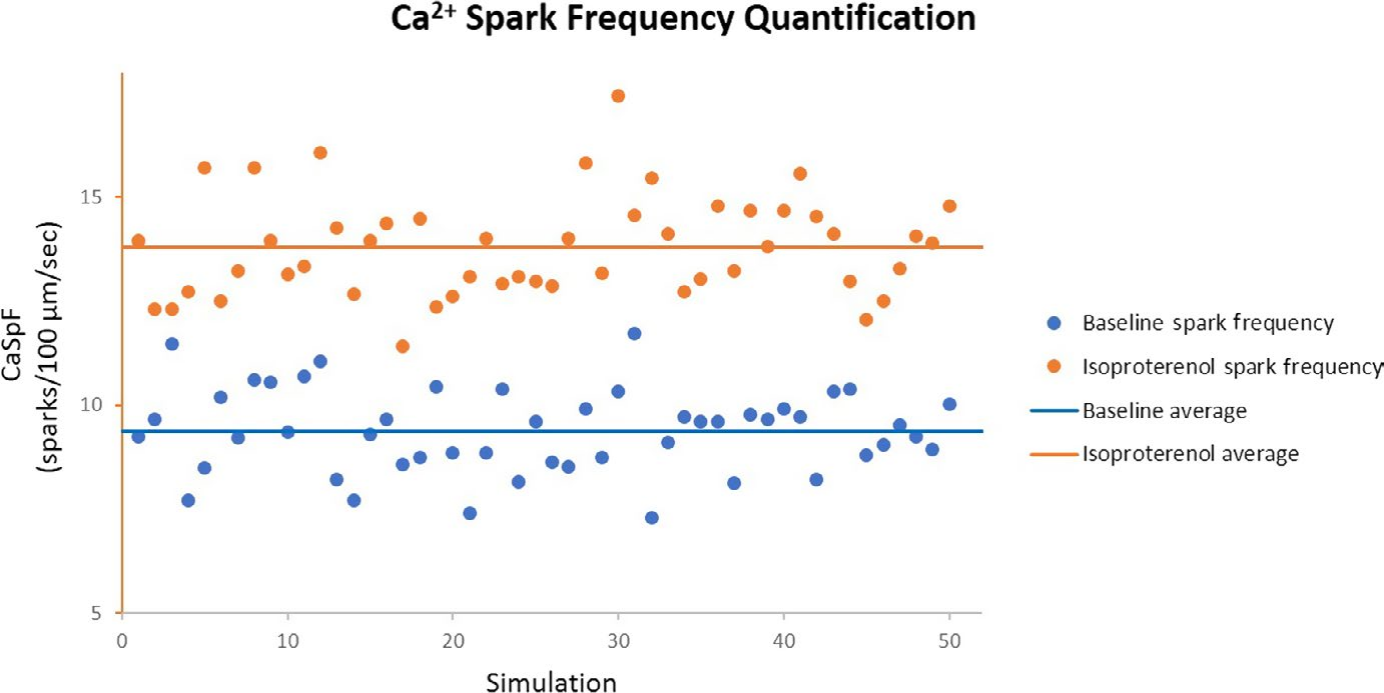
Scatter plot of Ca^2+^ spark frequencies for 50 simulations in the CPVT‐phenotype PC model. Blue and orange circles are the spark frequencies for individual simulations in the absence and presence of isoproterenol, respectively. Both baseline and isoproterenol models were burst paced for 5 s at 5 Hz stimulation and then observed for 15 s postpacing

We further quantified the Ca spark frequency obtained in the CPVT model. Figure 7 shows a scatter plot of Ca spark frequencies obtained in 50 simulations with and without isoproterenol. The model was paced at 5 Hz for 5 s, after which Ca sparks were quantified during 15 s postpacing. Our model yielded average spark frequencies of 9.38 ± 1.00 and 13.78 ± 1.21 per 100 µm/s without and with isoproterenol, respectively, which are comparable with the experimental results in transgenic mouse PCs (Stuyvers et al., 2005).

### 3.5 Effects of ion channel blockade on CPVT model

**Figure 8:**
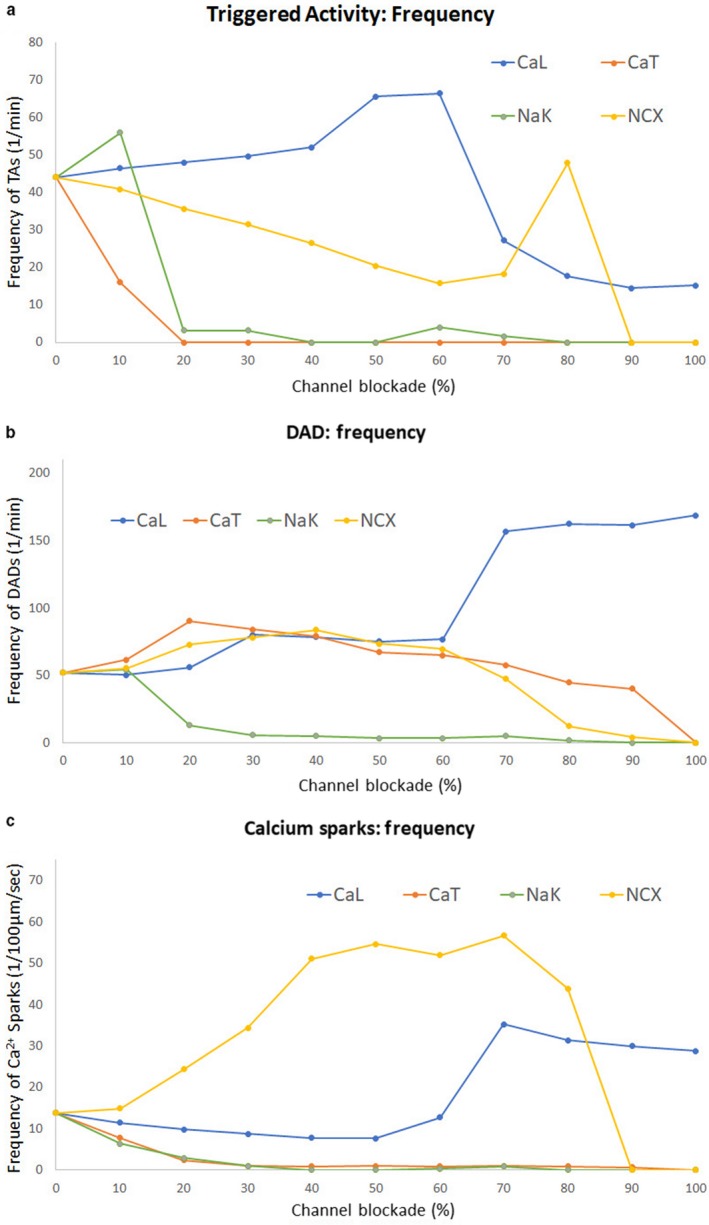
(a) Frequency of triggered activity, (b) frequency of DADs, and (c) frequency of Ca^2+^ sparks as L‐type Ca^2+^ (CaL) channel, T‐type Ca^2+^ (CaT) channel, sodium‐potassium pump (NaK), and sodium‐calcium exchanger (NCX) are individually blocked. All simulations are in the presence of isoproterenol for the CPVT‐phenotype PC model

Finally, we examined the effects of single ion channel blockade on triggered activity, DADs and Ca spark frequency in the CPVT model in the presence of isoproterenol (Figure 8). Simulations for all individual blockades were allowed to reach steady state over 120 s prior to performing analyses. Ion channel blockade was conducted in intervals of 10% up to 100% on the following four channels: L‐type Ca channel (CaL), T‐type Ca channel (CaT), sodium‐potassium (NaK) pump and sodium‐calcium exchanger (NCX). Figure 8a shows the change in the frequency of triggered activity as each of the individual channels were blocked.

The most effective channel blockade in reducing triggered activity is the T‐type Ca channel. A 20% reduction in CaT completely eliminated triggered activity. Blockade of NaK above 20% also significantly reduced triggered activity. While NCX and I_CaT_ play a crucial role in DADs and DAD‐induced triggered activity, blocking NCX alone did not terminate the triggered activity until a 90% blockade in NCX, which highlights the importance of I_CaT_ in DAD‐induced triggered activity in PCs.

Interestingly, blockage of CaL, up to 60%, resulted in an increase in triggered activity. The resulting dynamics following CaL blockage resulted in a net increased average inward current, calculated over the 15 s post burst pacing, through the fast sodium channel, as seen in Figure S4a. Increased inward current through the fast sodium channel promoted depolarization of the PC and increased excitability. Similarly, an increase in triggered activity was observed when NCX was blocked by 80%. Figure S4b shows the sharp increase in average inward current through the fast sodium channel for 80% NCX blockage, similarly enhancing depolarization of the PC. Higher percentages of blockade of all channels decreased triggered activity.

While triggered activity was diminished in most cases as channel blockade increased, the frequency of DADs did not follow a similar pattern (Figure 8b). For lower percentages of blockade, only the blockade of NaK successfully decreased the DAD frequency. DAD frequency did not change significantly for CaL, CaT and NCX blocked up to 60%. For 70% and greater blockage of CaT and NCX independently, DAD frequency decreased. However, 70% and greater blockade of CaL resulted in increased DAD frequency, though these DADs did not drive triggered activity (Figure 8a). Lastly, Ca spark frequencies were analyzed as ion channels were blocked (Figure 8c). Increasing blockade of CaT and NaK resulted in decreased Ca spark frequencies. Interestingly, blockage of NCX resulted in a large increase in Ca sparks, which rapidly decreased after 80% blockade. Additionally, while low percentage of CaL blockade decreased Ca spark frequency, 70% and greater blockade resulted in an elevated and sustained increase in Ca sparks.

We further investigated the influence of L‐type Ca channel blockage and found that as the blockade of L‐type Ca channel increased, Ca release from the JSR decreased during spontaneous Ca leaks, DADs and triggered activity (Figure S5). The average JSR [Ca] was calculated over 15 s post burst pacing for 5 s at 5 Hz, and we found that the mean JSR [Ca] increased with increasing blockade of the L‐type Ca channel, in particular at 70% and higher blockade (Figure S6). Thus, for 70% and above CaL blockage, the elevated JSR [Ca] drives an increase in Ca spark frequency.

We performed further analysis to understand the increase in Ca spark frequency with increasing NCX blockade. We analyzed currents through other channels as NCX blockade was increased. We found that as NCX blockade was increased (up to 80%), there was an increase in inward Ca current through the T‐type Ca channel (Figure S7). Figure S5 shows that as NCX blockage increases, sub‐SR Ca increases, due to Ca sparks, and there is an associated inward Ca current increase through the T‐type Ca channel. Quantification of the increase in inward Ca currents through the T‐type Ca channel is provided in Figure S8. As NCX blockade increases, the average duration of increased inward Ca currents through CaT increases in a similar pattern to the increase in Ca spark frequency (Figure S8a). A similar pattern is seen for the total duration of increased inward CaT current as NCX blockade is increased (Figure S8b). Of note, the amplitude of increased inward current through CaT is not significantly changed as NCX blockade increases (Figure S8c).

## 4 DISCUSSION

In this study, we employed a detailed stochastic modeling approach to investigate the mechanisms of DAD generation and triggered activity in cardiac Purkinje cells mediated through cytosolic Ca diffusion. Our main findings are summarized as follows: (a) Ca diffusion produced biphasic cytosolic Ca transients, namely, radial wavelets and longitudinal CWWs; (b) SCRs caused by leaky RyR channels can initiate reverse direction Ca waves propagating from the core of the cell to the sarcolemma; (c) Elevated Ca concentration in the sub‐SL region due to SCRs may reactivate currents from the T‐type Ca channels and sodium‐calcium exchanger, which result into membrane depolarizations in the form of DADs; (d) Excessive membrane depolarizations due to frequent SCRs can activate inward sodium channels thereby producing DAD‐induced triggered activity; and (e) In the presence of isoproterenol in our model, activation of either I_CaL_, I_NaK,_ I_CaT_ or NCX is sufficient for occurrence of the triggered activity; however, a partial blockade of either I_NaK_ or I_CaT_ is essential for its successful termination.

### 4.1 Calcium diffusion waves in PCs

The Ca release units are spatially correlated with the T‐tubule structure in ventricular myocytes. However, the lack of well‐defined T‐tubules in PCs makes it challenging to determine the spatial distribution of RyR channels. As such, no experimental data are available on the subcellular localization and spatial distribution of Ca release sites in PCs. Hence, we modeled the wave‐like CICR propagation of Ca into the cell interior, similar to other modeling approaches in atrial myocytes (Thul, Coombes, Roderick, & Bootman, 2012; Voigt et al., 2014). Stochastic gating of RyR channels reproduced the experimentally recorded probabilistic behavior of SR Ca release (Rüdiger et al., 2007; Weinberg & Smith, 2012). Further, this modeling framework reproduced the biphasic cytosolic Ca waves observed in PCs, with radial and longitudinal cell‐wide components (Stuyvers et al., 2005; Ter Keurs & Boyden, 2007). We implemented longitudinal Ca waves using diffusion between 10 subcellular discs with individual Ca release and uptake compartments. Thus, we were able to achieve cell‐wide Ca waves by triggering CICRs of individual discs as the wave propagates throughout the cell. Ca wavelets, on the other hand, are propagated as simple diffusion between the cell periphery and the core of the cell. The combined effect of both radial wavelets and longitudinal cell‐wide Ca waves produced the experimentally recorded biphasic Ca transients. Further, cell‐wide waves traveled at a constant velocity and amplitude, an indication of contributions from local CICRs (Ter Keurs & Boyden, 2007) which has also been demonstrated by experiments in PCs using thapsigargin and ryanodine as agents to alter the CICR dynamics (Boyden et al., 2000).

### 4.2 Role of Ca2+ waves in DADs and triggered activity

It has been suggested that the intracellular SCR events from the SR caused by store overload‐induced Ca release (SOICR) (Jiang et al., 2005) may initiate Ca waves and cause a dramatic increase in cytosolic Ca (Priori & Chen, 2011). Such increase in cytosolic Ca may produce DADs due to transient inward current mediated through NCX (Lakatta, 1992; Marban, Robinson, & Wier, 1986). PCs also exhibit T‐type Ca channels which activate at lower membrane potentials (−70 to −60 mV) (Vaidyanathan et al., 2013). These channels have been shown to contribute to the diastolic depolarization and triggering of an AP in pacemaking cells (Hüser, Blatter, & Lipsius, 2000; Imtiaz, Weid, Laver, & Helden, 2010). T‐type channels have also been implicated in the pathogenesis associated with Ca overload and cardiomyopathy‐related arrhythmias (Chiang et al., 2009; Martínez, Heredia, & Delgado, 1999). In our simulations, we observed that I_CaT_, in conjunction with NCX‐mediated current, contributed to DAD generation and played a major role in increased propensity to DADs in RyR2‐mutated PCs as observed in experiments (Pallante et al., 2010; Ter Keurs & Boyden, 2007) and other recent modeling studies (Li & Rudy, 2011; Ten Tusscher & Panfilov, 2008).

Consistent with prior studies, (Pogwizd & Bers, 2004; Schlotthauer & Bers, 2000) our simulations showed that if the magnitude of DADs sufficiently reaches the activation potential of fast inward sodium channels (I_Na_), a triggered AP is elicited. We observed that activation of I_CaT_ and/or NCX was sufficient for the DAD‐induced triggered activity in our PC model, but blockade of I_CaT_ or I_NaK_ was essential to successfully terminate triggered APs. We observed that the presence of I_CaT_ produced diastolic depolarizations that were sufficient to reach the activation threshold of I_Na_ resulting in triggered activity, in contrast with I_CaL_. This could explain why blockade of I_CaT_ resulted into termination of the triggered activity in our simulations. Interestingly, I_CaT_ is absent in VMs (Niwa et al., 2004) which may explain why only PCs exhibited DAD‐induced triggered activity under beta‐adrenergic stimulation in RyR2‐mutated mice, (Cerrone et al., 2007; Fernández‐Velasco et al., 2009; Herron et al., 2010) although both VMs and PCs are capable of producing DADs. DADs may be readily generated in isolated VMs, however, in well‐coupled ventricular tissue, the electrotonic currents flow to the neighboring cells which greatly reduces the DAD amplitude of the source cell and suppresses the possibility of DAD‐induced triggered activity (Xie, Sato, Garfinkel, Qu, & Weiss, 2010). Indeed, in order to successfully trigger a premature AP in ventricles, a large number of adjacent myocytes need to generate a DAD within sufficiently small window in time (Campos et al., 2015; Campos, Shiferaw, Vigmond, & Plank, 2017). In the Purkinje system, the electrotonic loading effect is minimal due to its 1D structure and hence it favors the first initiation of DAD‐induced triggered activity (Campos et al., 2015; Deo et al., 2010). The presence of well‐established T‐tubule structure in VMs enables them to generate whole‐cell Ca transients. In contrast, the PCs do not express an extensive T‐tubule structure and hence rely mainly on Ca channels at the periphery. This leads to a very distinct systolic Ca signaling mediated via cytosolic Ca diffusion waves in PCs. Our numerical investigation showed critical mechanisms of arrhythmia that were initiated by the reverse direction cytosolic Ca diffusion waves. More simulations are warranted in future using PC models with well‐established T‐tubule structure versus nonexistent T‐tubules to establish the mechanistic differences in Ca homeostasis and its implications on arrhythmogeneity.

### 4.3 Limitations

In our study, we used 5–20 Hz burst pacing to load the SR with Ca to study the occurrences of DADs and triggered activity in presence of isoproterenol which has been a widely used experimental protocol. However, it should be noted that considering the physiological heart rates in mice, which range from 5–13 Hz, our pacing rates are on the lower side. It has been reported that there are three types of Ca channels in PCs, associated with IP_3_R_1_, RyR_2_ and RyR_3_ receptors, that cause the biphasic Ca transients. Although our model does not differentiate between these receptor types, our aim was not to study the mechanism of biphasic Ca transients, but rather to study the arrhythmogenic effects of Ca waves produced by the biphasic transients.

## 5 CONCLUSIONS

We present a systematic in silico study to understand the mechanisms of DAD generation and DAD‐induced triggered activity using a morphologically realistic numerical model of a PC capable of simulating spatiotemporal Ca waves. We found that the spontaneous Ca leaks from SR as a result of RyR mutations may produce Ca waves that are capable of depolarizing the cell membrane via activation of inward NCX and I_CaT_. As the frequency and magnitude of SCR events increase, DADs were converted into triggered activity via reactivation of inward I_Na_. Although NCX and I_CaT_ both play a major role in DAD‐induced triggered activity, blockade of I_CaT_ was essential for successfully terminating the triggered activity. Our study provides useful insights into the role of cardiac Purkinje system in initiation of arrhythmia in inherited syndromes and potential therapeutic targets for Purkinje system‐mediated arrhythmias.

## CONFLICT OF INTEREST

The authors have no competing interests related to the study presented in this manuscript.

## AUTHOR CONTRIBUTIONS

C.S. performed the modeling and simulations, and wrote the first draft of the manuscript. S.J. and B.L. developed the initial model formulations and design. S.W. reviewed the work and made critical revisions to the manuscript. M.D. conceived the design of the study, made critical revisions to the manuscript and approved the final version.

## Data Availability

The numerical modeling data generated during and/or analyzed during the current study are available from the corresponding author on reasonable request.
