# Supplementary material for: Delayed afterdepolarization‐induced triggered activity in cardiac purkinje cells mediated through cytosolic calcium diffusion waves
Source: Physiol Rep. 2019 Dec 23;7(24):e14296. doi: 10.14814/phy2.14296 (PMC6928245; doi:10.14814/phy2.14296)
Supplement: Supplementary file 1 [file PHY2-7-e14296-s001.pdf]

Delayed Afterdepolarization-induced Triggered Activity in Cardiac Purkinje Cells Mediated through Cytosolic Calcium Waves

Authors: Chirag Shah, Sohel Jiwani, Bijay Limbu, Seth Weinberg, Makarand Deo*

Supplemental Figures

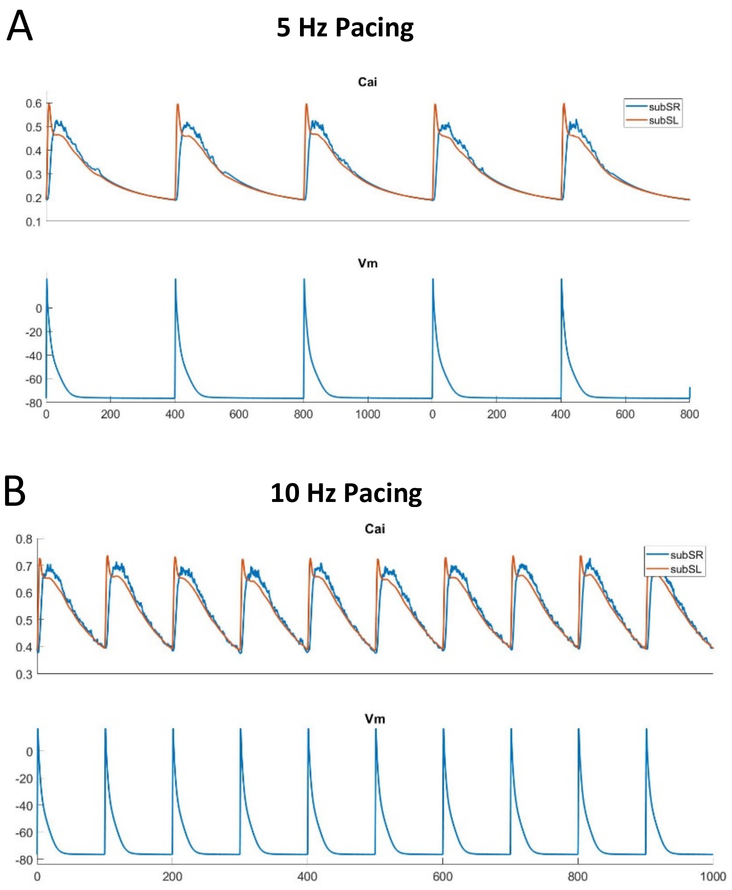

Supplemental Figure 1. Intracellular Ca transients in subSR and subSL (top) and action potentials (bottom) elicited at pacing frequencies of **(A)** 5Hz, and **(B)** 10 Hz.

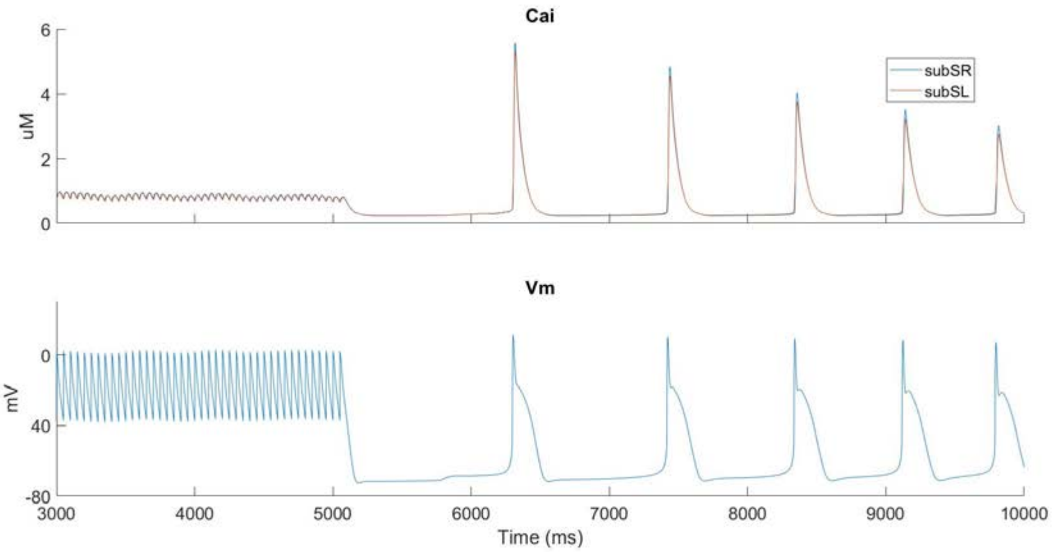

Supplemental Figure 2. Spontaneous Ca release (top) and triggered action potentials (bottom) after 20 Hz burst pacing in presence of isoproterenol.

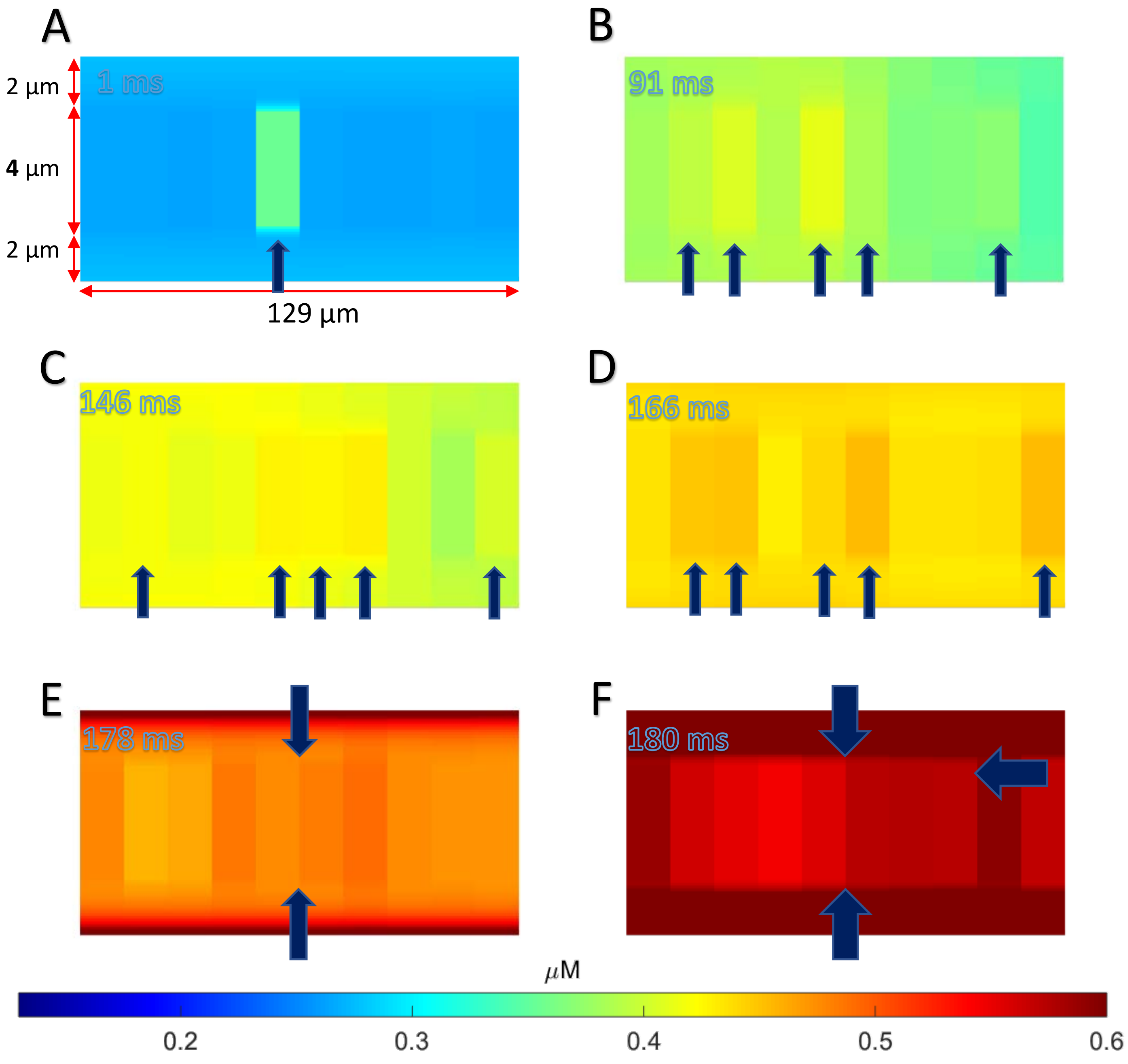

Supplemental Figure 3. Ca^{2+} spatial plots illustrating Ca^{2+} sparks during DAD-induced triggered activity in the CPVT phenotype model with isoproterenol stimulation. Arrows in **(A) – (D)** indicate SR discs where Ca^{2+} sparks are occurring at the specific time. Arrows in **(E)** and **(F)** show propagation of increasing Ca^{2+} concentrations once triggered activity is initiated.

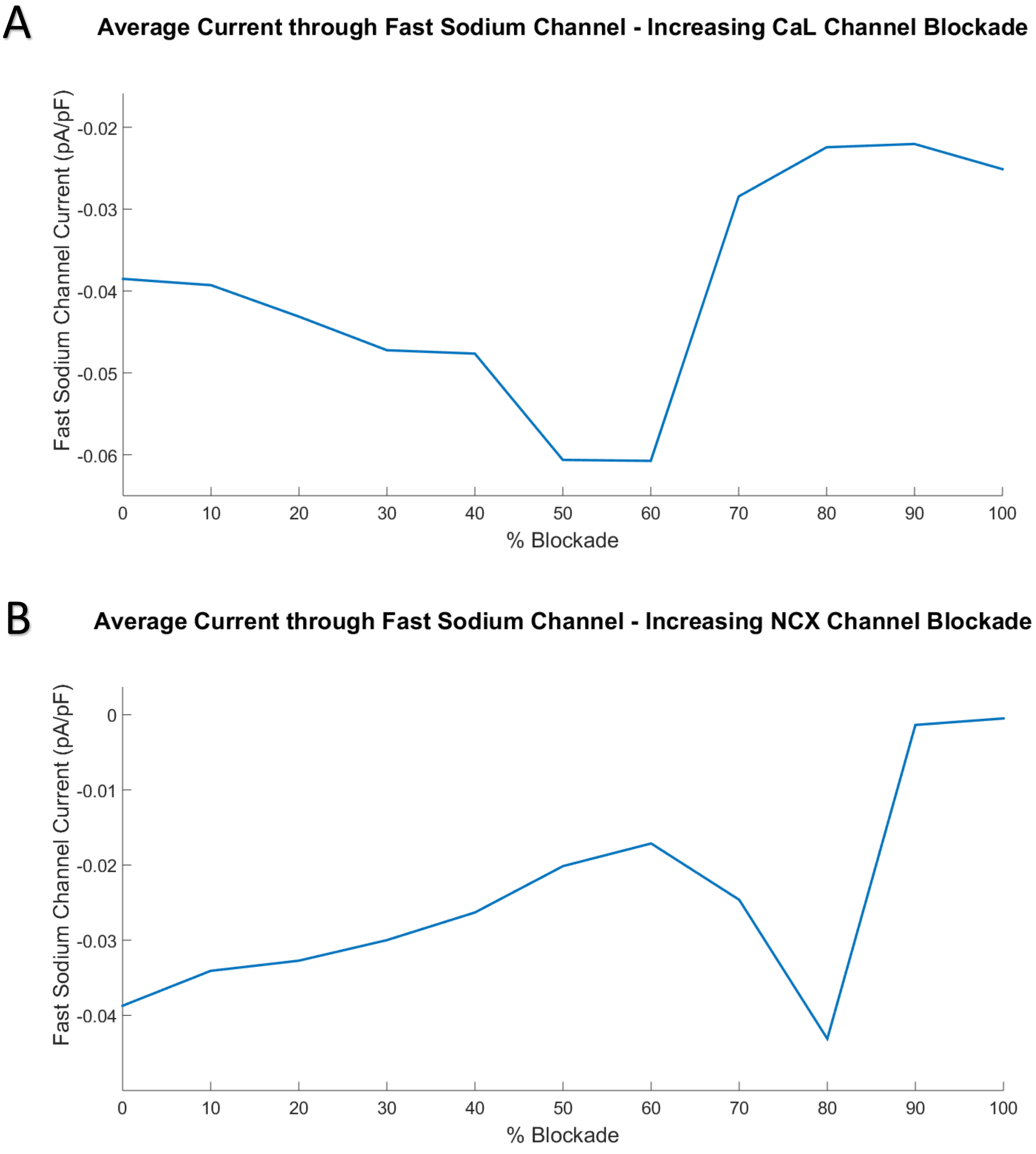

Supplemental Figure 4. (A) Current through fast sodium channel as L-type calcium channel is increasingly blocked. **(B)** Current through fast sodium channel as sodium-calcium exchanger is increasingly blocked. Traces in both figures are from simulations modeling the RyR2 mutations in the presence of isoproterenol.

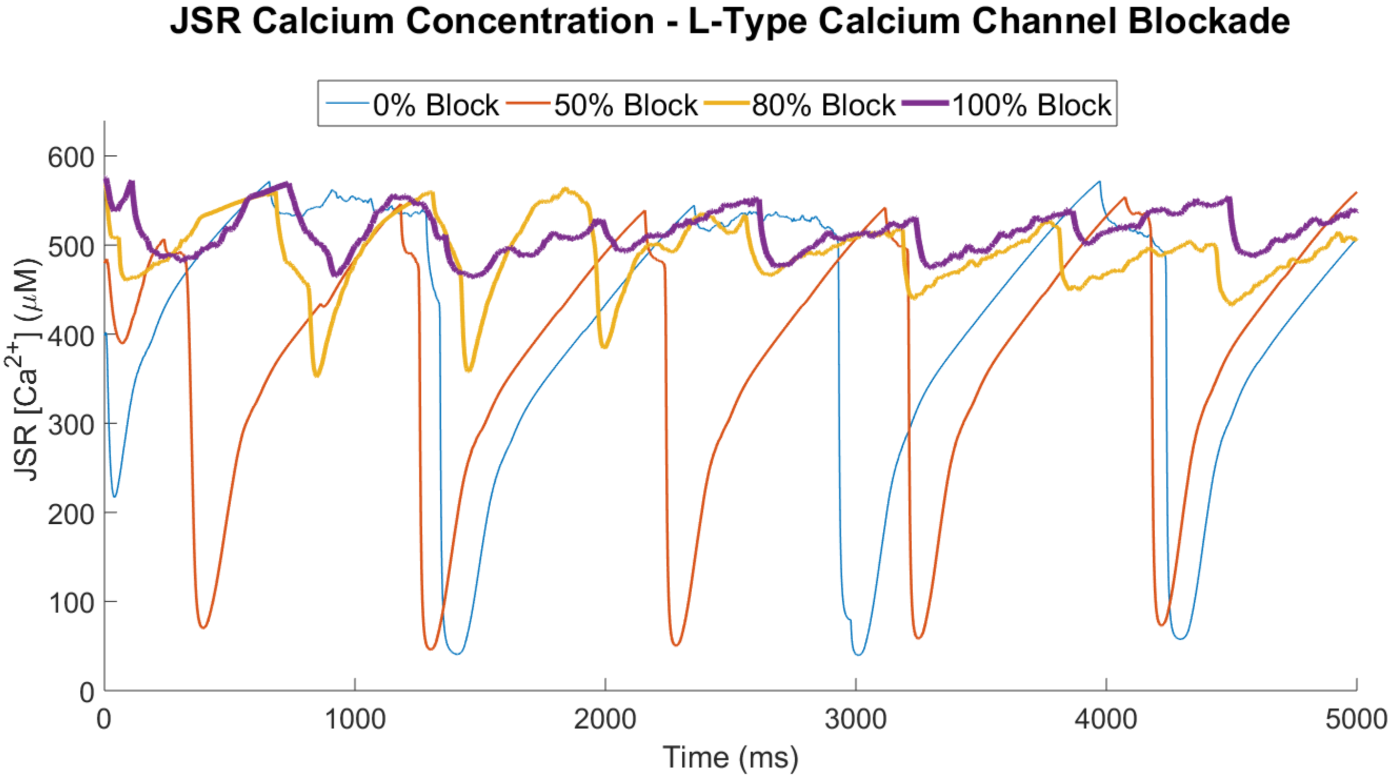

Supplemental Figure 5. Ca^{2+} concentration plots within the JSR with varying percentages of L-type Ca^{2+} channel blockade over 5 seconds post burst-pacing at 5 Hz. Traces in this figure are from simulations modeling the RyR2 mutations in the presence of isoproterenol.

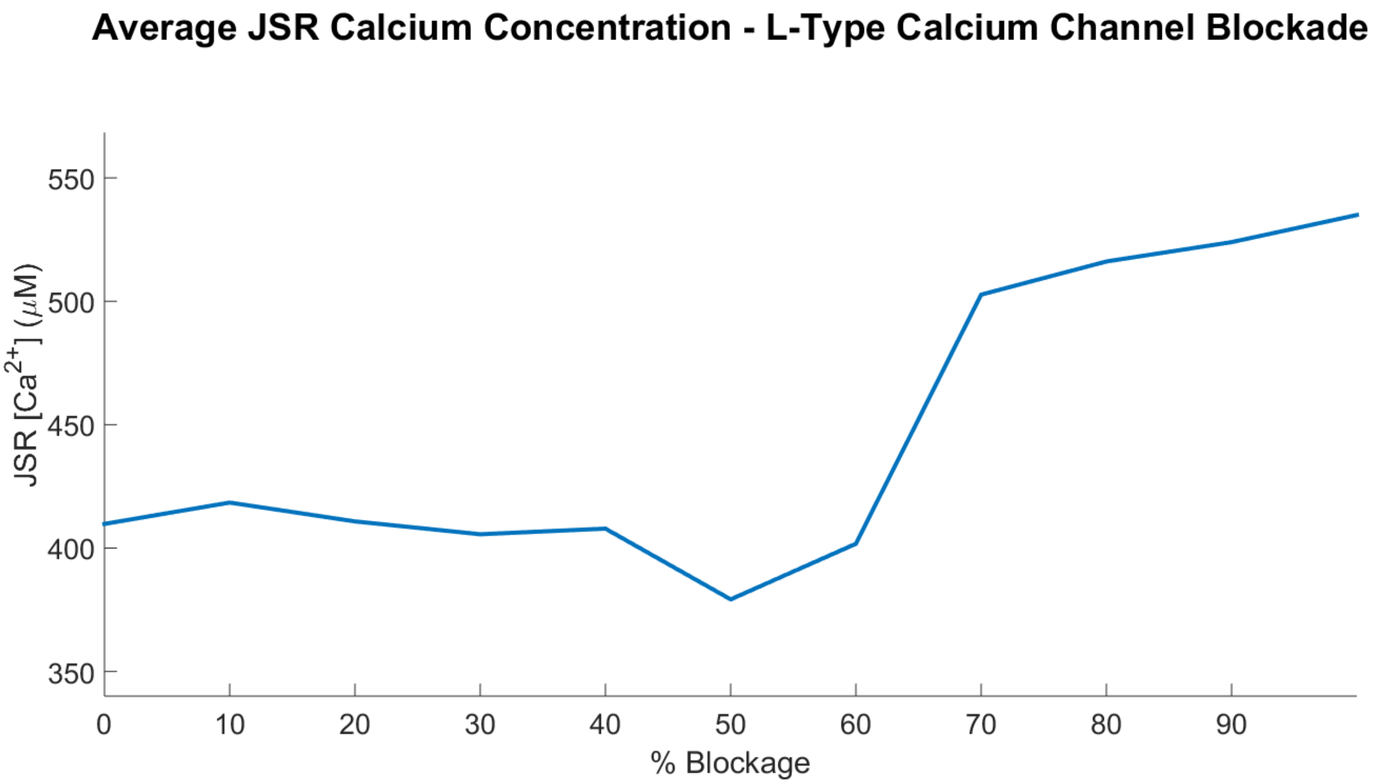

Supplemental Figure 6. Change in average [Ca^{2+}] concentration in JSR as L-type Ca^{2+} channel is increasingly blocked. Average [Ca^{2+}] concentration measured over 15 seconds post burst-pacing for 5 seconds at 5 Hz. Trace in this figure is from simulations modeling the RyR2 mutation in the presence of isoproterenol.

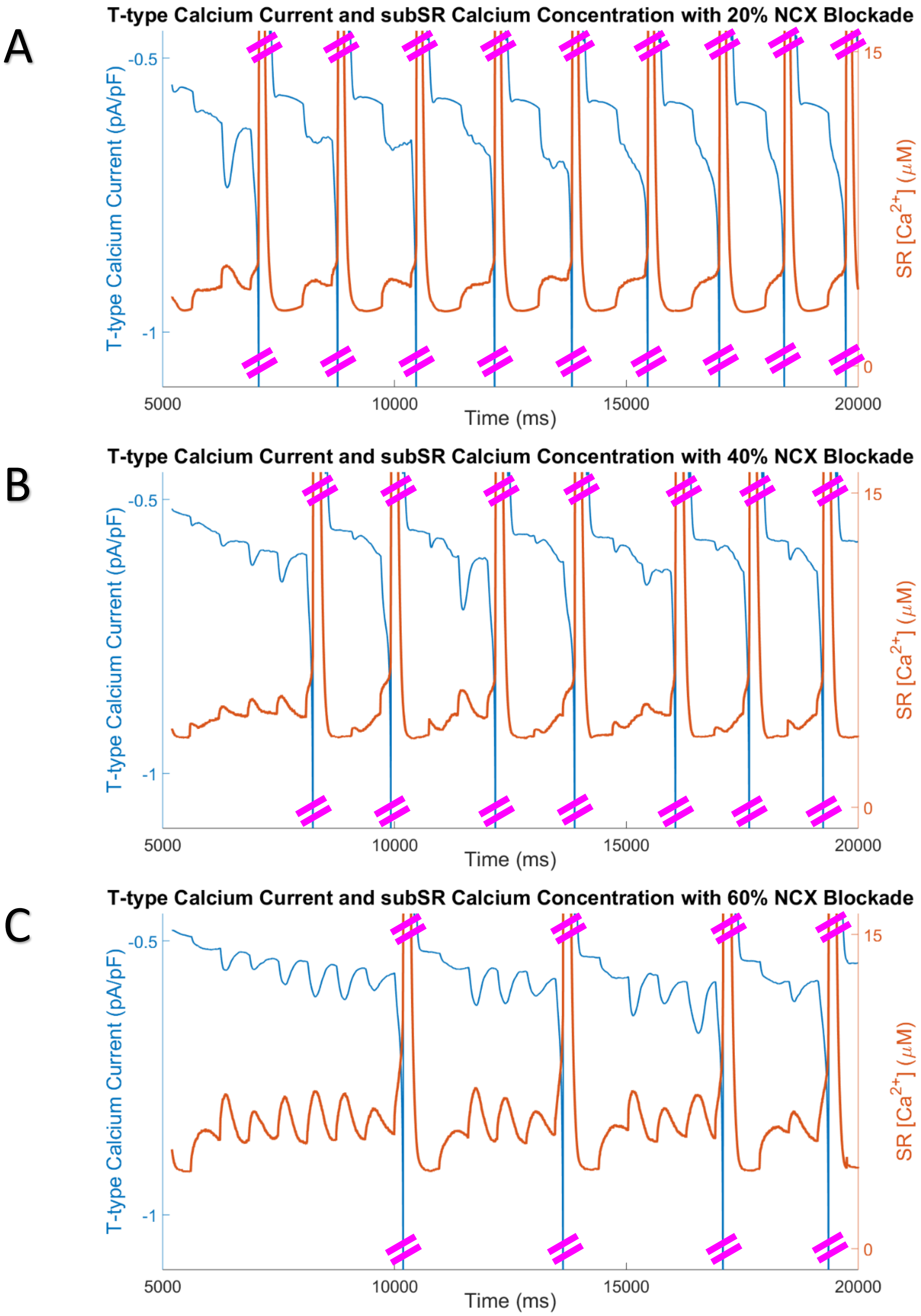

Supplemental Figure 7. T-type calcium channel current and [Ca^{2+}] concentration at SR with **(A)** 20% NCX blockade, **(B)** 40% NCX blockade, **(C)** 60% NCX blockade. Traces in these figures are from simulations modeling the RyR2 mutation in the presence of isoproterenol and show results after 5 seconds of burst pacing at 5 Hz.

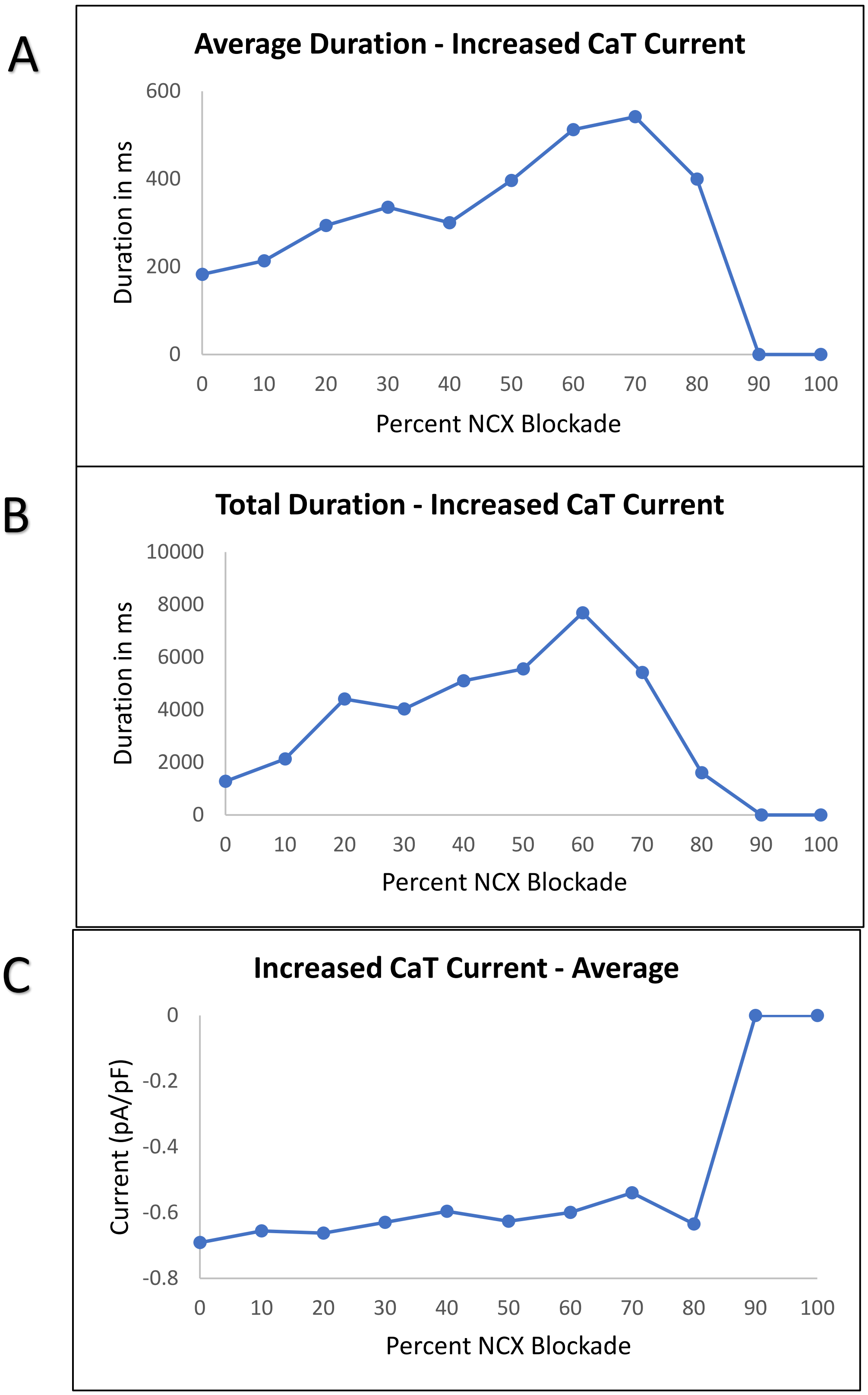

Supplemental Figure 8 (A) Average duration, **(B)** total duration, and **(C)** average current of all increases in inward current through the T-type Calcium channel, excluding during triggered activity, as NCX is increasingly blocked. Traces in these figures are from simulations modeling the RyR2 mutation in the presence of isoproterenol and show results measuring the currents 15 seconds after 5 seconds of burst pacing at 5 Hz.
